# Breast Cancer Genetic Services in a South African Setting: Proband Testing, Cascading and Clinical Management

**DOI:** 10.1002/cam4.70743

**Published:** 2025-03-05

**Authors:** T. S. Osler, M. Schoeman, J. Edge, W. J. S. Pretorius, F. H. Rabe, C. G. Mathew, M. F. Urban

**Affiliations:** ^1^ Sydney Brenner Institute for Molecular Bioscience, Faculty of Health Sciences University of the Witwatersrand Johannesburg South Africa; ^2^ Division of Human Genetics, National Health Laboratory Service and School of Pathology Faculty of Health Sciences, University of the Witwatersrand Johannesburg South Africa; ^3^ Division of Molecular Biology and Human Genetics Faculty of Medicine and Health Sciences, University of Stellenbosch and Tygerberg Hospital Cape Town South Africa; ^4^ Department of Surgery Charlotte Maxeke Johannesburg Academic Hospital and University of the Witwatersrand Johannesburg South Africa; ^5^ Faculty of Medicine and Health Sciences University of Stellenbosch and Tygerberg Hospital Cape Town South Africa; ^6^ Department of Obstetrics and Gynaecology Faculty of Medicine and Health Sciences, University of Stellenbosch and Tygerberg Hospital Cape Town South Africa

**Keywords:** *BRCA1/2*, cancer prevention, cascade testing, genetic testing

## Abstract

**Purpose:**

Limited data exist on managing hereditary breast cancer in low‐ middle‐income countries (LMICs). We assessed proband and cascade genetic testing, and risk‐reducing measures in a South African regional breast cancer service.

**Methods:**

We analysed records from 534 consecutive female probands receiving genetic counselling for breast cancer and their 115 relatives who attended genetic counselling. Demographic and clinical data, family pedigrees and genetic test data were collated from hospital clinical records, regional laboratory data, screening appointments and radiological records.

**Results:**

Test uptake in probands was high (86.9%), although cost was a deterrent in some. There were 83 (19.6%) probands who tested positive, and 45.0% of them had one or more family members have testing. This resulted in 9.2% of relatives (first‐ to third‐degree) having cascade testing. Family testing was associated with a stronger family history of cancer, female gender and being a first‐degree relative (uptake was 25.6% in female first‐degree relatives). Risk‐reducing mastectomy was accepted by 52.6% of eligible female family members, while mammographic surveillance (30%) and bilateral salpingo‐oophorectomy (15.4%) were less frequent.

**Conclusion:**

Genetic testing was well accepted by probands, but uptake was low in family members. Overall, one family member carrying a pathogenic variant was identified for every 13 probands receiving genetic counselling and for every 11 probands tested. Risk‐reducing measures were taken up by over half of those eligible. Limited uptake of cascade testing and variable uptake of risk‐reducing options impacted the programme. To our knowledge, this is the first study in Africa of the real‐world effectiveness of a breast cancer genetic service.

## 1 Introduction

Breast cancer (BC) is the commonest cancer in South African women, with prevalence estimates from the National Cancer Registry of 1 in 43 for Black African women, 1 in 18 for women of Mixed Ancestry, and 1 in 10 for White women [1]. Internationally, 5%–10% of unselected cases of BC are attributable to a pathogenic or likely pathogenic (P/LP) variant in a BC susceptibility gene [2, 3]. Identifying these individuals through genetic counselling and testing (GCT) allows the implementation of recommended treatment modifications and risk‐reducing strategies [4].

Although GCT is the standard of care in high‐income countries (HIC), it is not widely available in low‐to‐middle‐income countries (LMIC) like South Africa [5]. Barriers to these services in LMIC include underfunded and fragmented health care services, as well as financial, geographical and logistical barriers [6, 7]. South Africa's healthcare system comprises a private sector, which primarily serves individuals with medical insurance, and a state‐provided sector, on which approximately 80% of the population relies [8]. GCT services are available within the state healthcare system at tertiary facilities in only three of the country's nine provinces. Medical costs, including those for GCT, are subsidised in state facilities on an income‐based sliding scale, ranging from full subsidies to none [9]. In South Africa, cancer genetic services are provided mainly by genetic counsellors ‐ nationally there are 28 practicing genetic counsellors, the majority of whom work in private practice. This number is estimated to represent just 5% of the genetic counselling workforce needed to meet the country's demands. In addition to the workforce shortage, there is a significant disparity in geographic distribution, with most genetic counsellors concentrated in urban centres [10]. Qualitative studies have shown that while many South Africans are unfamiliar with genetic counselling, they generally respond positively to the process after experiencing it [11, 12].

Identifying individuals with a P/LP variant in a BC susceptibility gene enables the use of risk‐reducing and surveillance measures for cancer recurrence and/or occurrence [4]. It is well established that risk‐reducing mastectomy (RRM) reduces the risk of BC by at least 90% in asymptomatic *BRCA1/2* carriers, while bilateral salpingo‐oophorectomy (BSO) significantly reduces morbidity and all‐cause mortality [13, 14, 15]. In addition, the National Comprehensive Cancer Network (NCCN) guidelines developed in the USA recommend surveillance for BC with MRI and/or mammogram starting from between 25 and 30 years of age. Similar evidence‐based guidelines are available for other BC susceptibility genes [4]. The uptake of risk‐reducing strategies has been shown to vary widely between countries [16], but little data is available from LMIC.

When an individual is identified to have a P/LP variant in a cancer susceptibility gene, cascade testing, the process of extending GCT to family members, can proceed. Offit et al. [17] calculated that if 70% of first, second‐ and third‐degree family members were to have testing, most individuals with a P/LP variant in the population would be identified within a decade. In practice, this has been difficult to realize even in HIC, with various barriers identified including contextual factors [18], poor family communication and/or relationships [19, 20], and others [21, 22, 23]. Currently, there is also little data on the uptake of cascade testing for hereditary cancer in LMIC, with two studies, both in research cohorts, showing divergent outcomes [24, 25].

Considering the dearth of data on GCT for inherited BC in South Africa and other LMICs, we aimed to assess its effectiveness in facilitating the pathway from proband to cascade genetic testing and risk‐reducing measures among family members in a regional BC service.

## 2 Methods

### 2.1 Study Site

This study was conducted at Tygerberg Academic Hospital (TAH), a tertiary hospital that serves as the regional cancer genetic referral centre for ~3.5 million people, 60% living in metropolitan Cape Town and the remainder in towns and farms up to 300 km away (see Figure S1). Within the region and during the study period, most aspects of BC care were provided at TAH, although breast surgery or cancer surveillance was also available at three associated secondary hospitals. Care was coordinated by a multidisciplinary team that included the breast surgery, oncology and anatomical pathology services, as well as a genetic counsellor based at TAH. Pre‐test genetic counselling was provided at TAH in person by a genetic counsellor or medical geneticist. Post‐test counselling, including the discussion of management options for clinically relevant genetic variants, was provided in person or telephonically.

### 2.2 Participants

Records were reviewed for female probands and their family members. Initial contact with the genetic service was almost always by a proband (a woman with invasive or in situ BC), following referral from the BC service. They were offered testing if the genetic health provider considered them eligible according to the South African National Department of Health (NDOH) guidelines [26]. Based on these guidelines, a woman with BC was eligible if she met any of the following criteria: diagnosis with BC at ≤ 40 years; two primary BCs or triple‐negative BC at ≤ 60 years; or a family history of either ≥ 1 close relative with invasive ovarian cancer, male BC, or BC at ≤ 50 years, or ≥ 2 close relatives with breast or pancreatic cancer, with one diagnosed ≤ 60 years. Probands were excluded if they had genetic testing prior to attending genetic counselling. Probands identified to have a P/LP variant in a BC susceptibility gene were asked to invite family members to attend GCT.

### 2.3 Study Period

Probands were included if they were seen for pre‐test GCT between 1 January 2018 and 31 August 2022. This timeframe was chosen because it was the period for which next generation sequencing (NGS) gene panel testing was available through the global testing platform of Invitae Corporation (San Francisco, USA) [27]. Family members were included if they attended GCT at TAH by 31 December 2022. To allow a minimum of a full year for implementation of risk‐reducing measures, medical records of both probands and family members were reviewed until 31 December 2023.

### 2.4 Genetic Testing

Of the probands tested, 86.8% had NGS gene panel testing through Invitae and 19.8% received testing through the South African National Health Laboratory Service (NHLS) (Table 3) [27, 28]. Testing by the NHLS was done using various methods: targeted analysis of 8 well‐documented founder mutations in *BRCA1/2* (see Table S1); single nucleotide and copy number variant (CNV) detection within the exons and splice‐site junctions of *BRCA1/2*; family variant testing; and an NGS panel of 15 genes. NHLS testing is conducted in an accredited diagnostic laboratory, with variants interpreted using standard guidelines [29, 30, 31].

The Invitae NGS gene panel protocol included assessment of single nucleotide variants and, for most genes, also copy number variants in the coding and flanking DNA, with variants interpreted using their Sherloc guideline [27, 32]. Family variant analysis performed by Invitae was available free of charge for 3 months via the TAH genetic counselling service to family members. By comparison, the cost of family variant analysis through the NHLS depended on patient eligibility for a financial subsidy; this also applied to both Invitae and NHLS proband tests.

In this study, we included only the results for 13 genes known to be associated with BC and having clinical guidelines for risk reduction (Table 1). Because not all probands had testing of the same genes, the denominators used to calculate the prevalence of P/LP variants differed.

**TABLE 1:** Genes assessed—number of probands tested and guidelines used to assess risk‐reducing interventions.

Gene	Included on NHLS panel [30]	Included on invitae panel [27]	Number of probands tested^a^	NCCN guideline starting age recommendations for risk‐reducing interventions		
				Mammo‐gram^b^	RRM	BSO
*ATM*	Yes	Yes	372	40 years	No	No
*BARD1*	Yes	Yes	313	40 years	No	No
*BRCA1*	Yes	Yes	393	25 years	25 years	35 years
*BRCA2*	Yes	Yes	393	25 years	25 years	40 years
*CDH1*	—	Yes	313	30 years	30 years	No
*CHEK2*	Yes	Yes	372	40 years	No	No
*NF1*	—	Yes	321	30 years	No	No
*PALB2*	Yes	Yes	372	30 years	30 years	45 years
*PTEN*	—	Yes	372	30 years	30 years	No
*RAD51C*	—	Yes	321	40 years	No	45 years
*RAD51D*	—	Yes	321	40 years	No	45 years
*STK11*	—	Yes	343	30 years	30 years	No
*TP53*	Yes	Yes	372	20 years	20 years	No

Abbreviations: BSO, bilateral salpingo‐oophorectomy; NCCN, National Comprehensive Cancer Network (USA); NHLS, National Health Laboratory Service (South Africa); RRM, risk‐reducing mastectomy.

^a^ Does not include probands who only had *BRCA1/2* founder variant (*n* = 29) or known family variant testing (*n* = 2).

^b^ Due to lack of access to MRI at TAH Hospital, only mammogram surveillance was analyzed.

### 2.5 Data Sources and Collection

Demographic, clinical, genetic counselling and testing data were collected for probands, and more limited data for family members. Five electronic data sources were used: NHLS and Invitae online portals for laboratory data, the TAH Enterprise Content Management system (ECM) (the hospital electronic archive of scanned clinical notes) for clinical data, the Picture Archiving and Communication System (PACS) for imaging results, and Clinicom for clinic appointments. Both Clinicom and PACS provided information on appointments and imaging results not only for TAH but also for secondary hospitals in the region, enabling a comprehensive assessment of screening performed across the area. Two supplementary sources of clinical information were hard copy records held by the Genetic Counselling service and the BC service. The variables collected from each data source are summarised in Table 2.

**TABLE 2:** Types and sources of data collected for probands and their family members at Tygerberg Academic Hospital (TAH).

Data source	Access (coverage)	Data type	Collected for probands	Collected for family members
TAH Enterprise Content Management (ECM system) [33]	Electronic (TAH)	Demographic data	Date of birth, gender, ethnicity, distance from TAH, financial subsidy level	Date of birth, gender
		Clinical data	BC histology, BC stage, age at BC diagnosis, any surgeries done	History of previous cancers, notes on RRM and/or BSO
		Family pedigree data	Number of FDR, SDR and TDR with cancer, family member age, family member age at cancer diagnosis	Relationship to proband
TAH Genetic Counselling Clinic records	Hard copy (Departmental)	Demographic data	To supplement data from electronic and online sources	To supplement data from electronic and online sources
		Genetic test data		
		Family pedigree data		
TAH Breast Surgery departmental records^a^	Hard copy (Departmental)	Clinical data	BC stage, breast surgery done, notes on BSO (if done)	RRM and/or BSO (if done)
Invitae online portal	Internet	Genetic test data	Result of Invitae genetic testing, ethnicity	Invitae genetic test result
NHLS reporting system (Trakcare)^b^	Internet (national)	Clinical data (histology records)	BC histology, BC stage, age at BC diagnosis, surgeries done	RRM and/or BSO (if done)
		Genetic test data	Results of genetic testing done at NHLS	Results of genetic testing done at NHLS
Picture Archiving and Communication (PACS) System	Electronic (regional reports)	Radiological procedures	Mammogram, breast ultrasound records	Mammogram, breast ultrasound records
Clinicom	Electronic (regional)	Appointment data	Attendance at screening mammogram and/or breast ultrasound	Attendance at mammogram and/or breast ultrasound

Abbreviations: BC, breast cancer; BSO, bilateral salpingo‐oophorectomy; FDR, first degree relative; NHLS, National Health Laboratory Service; RRM, risk reducing mastectomy; SDR, second degree relative; TDR, third degree relative.

^a^ Available from January 2021.

^b^ Laboratory reports from tests done in the state system countrywide.

Ethnicity was inferred by the genetic health professional and recorded on request forms, as required by testing laboratories, and categorised according to South African National Census groups: Black African, White, South African Coloured (Mixed Ancestry in this study) and Indian/Asian [34]. The Black African population is Bantu‐speaking peoples indigenous to Southern Africa, accounting for 38.8% of the Western Cape (WC) population [35, 36]. White South Africans, accounting for 16.4% of the WC population, are predominantly descendants of European settlers [36, 37]. People of Mixed Ancestry have genetically admixed ancestry, including Khoisan, Black African, European and Asian population roots [35, 38]. They form the largest population group in the WC, accounting for 42.1%. The Indian/Asian population represents a small minority (1.1%) of the WC population [36].

The financial subsidy provided to individuals for state healthcare, including genetic testing, is determined through a means‐tested system [9]. Patients with very limited financial means receive almost full subsidies for the direct costs of healthcare, including genetic testing and cancer management, while those with greater financial means receive only partial subsidies or none at all. There are four levels of financial subsidy (H0, H1, H2, H3), as detailed in Table 3. However, due to the smaller number of patients in the H1, H2 and H3 categories, we combined H0 with H1 and H2 with H3 for the purposes of comparison.

**TABLE 3:** Demographic and clinical data for study participants.

	Probands offered testing (*n* = 488)	Tested probands (*n* = 424)	Tested family members (*n* = 111)
Age at testing			
Median	41	41	35
Range	18–76	18–76	3–66
Unknown	0	0	0
Gender			
Female	488	424	87 (78.4%)
Male	0	0	24 (21.6%)
Distance from hospital			
< 50 km	379 (77.7%)	322 (75.9%)	30 (27.0%)
50–100 km	57 (11.7%)	53 (12.5%)	2 (1.8%)
100–200 km	41 (8.4%)	39 (9.2%)	0
> 200 km	10 (2.0%)	9 (2.1%)	2 (1.8%)
Unknown	1 (0.2%)	1 (0.2%)	77 (69.4%)
Ethnicity/Population group			
Mixed Ancestry	254 (52.0%)	237 (55.9%)	38 (34.2%)
Black African	100 (20.5%)	91 (21.5%)	25 (22.5%)
White	70 (14.3%)	61 (14.4%)	24 (21.6%)
Unknown	64 (13.1%)	35 (8.3%)	24 (21.6%)
Financial subsidy^a^ for GCT			
Full subsidy of consult and testing (H0)	382 (78.3%)	368 (86.8%)	21 (18.9%)
Subsidised fee covering consult and testing (H1)	76 (15.6%)	41 (9.7%)	2 (1.8%)
Subsidised consult fee and 20% of test cost (H2)	11 (2.3%)	5 (1.2%)	1 (0.9%)
No subsidy–pay consult and test cost (H3 or Pvt)	18 (3.7%)	9 (2.1%)	5 (4.5%)
Unknown	1 (0.2%)	1 (0.2%)	82 (73.9%)
Breast cancer diagnosis			
Yes	488 (100%)	424 (100%)	7 (6.3%)
No	0	0	102 (91.9%)
Unknown	0	0	2 (1.8%)
Breast cancer histology			
DCIS^c^	7 (1.4%)	6 (1.4%)	
Invasive ductal	418 (85.7%)	387 (91.3%)	
Invasive lobular	17 (3.5%)	15 (3.5%)	
Unknown	46 (9.4%)	16 (3.8%)	7^e^
Cancer stage^b^			
In situ	7 (1.4%)	6 (1.4%)	
Stage 1	28 (5.7%)	24 (5.7%)	
Stage 2	158 (32.4%)	141 (33.3%)	
Stage 3	194 (39.8%)	178 (42.0%)	
Stage 4	70 (14.3%)	61 (14.4%)	
Recurrence	5 (1.0%)	4 (0.9%)	
Unknown	26 (5.3%)	10 (2.4%)	7
Genetic tests done^c^			
8 founder variants^d^ (*BRCA1/2*)		50 (11.8%)	
*BRCA1/2* sequencing		22 (5.2%)	
NHLS panel test		8 (1.9%)	
Invitae panel test		368 (86.8%)	
Family variant testing		4 (0.9%)	111 (100%)

Abbreviations: DCIS, ductal carcinoma in situ; GCT, genetic counselling and testing.

^a^ Subsidy levels are determined by the Department of Health based on income [9].

^b^ Clinical breast cancer stage was determined by the tumour‐node‐metastasis (TNM) system, as indicated or inferred from the clinical notes.

^c^ The number of tests done (452) exceeds the number of tested probands (424), because > 1 test was done in some cases.

^d^ South African founder variants are listed in the Table S1.

^e^ 7 family members had breast cancer prior to testing.

Probands and family members were considered eligible for interventions based on the NCCN guidelines (Table 1). Surgeries such as mastectomies and BSOs performed in the state healthcare system should result in both a clinical note and a NHLS histology report. Mammogram and breast ultrasound screening required an electronic appointment on the Clinicom system and were reported in the Picture Archiving and Communication System (PACS) database. Therefore, for both surgical interventions and screening procedures, two sources of evidence were assessed. In addition, screening was reviewed for all probands and family members, except in cases where an RRM had already been performed. This is because RRM is routinely preceded by breast screening, after which patients are discharged. Chemoprevention is not available for cancer‐unaffected women in the South African state system and was therefore not assessed.

### 2.6 Data Analysis

All statistical analysis was done using RStudio version 2024.4.0 [39]. and *p* < 0.05 was considered statistically significant. Univariate logistic regression was used to compare the distribution of both categorical and continuous variables for the uptake of genetic and family member testing. Factors with *p* < 0.2 were then included in a multivariate logistic regression analysis for uptake of genetic testing in probands. Highly correlated factors, determined using variance inflation factors, were dropped from the model. As only one variable was found to have *p* < 0.2 when factors impacting test uptake in family members were examined, multivariate analysis was not required. Results are reported as odds ratios (OR) with 95% confidence intervals (CI) and associated *p*‐values.

Family pedigrees were used to determine the number of family members eligible for cascade testing. Family members were not considered eligible if < 18 years unless the familial P/LP variant was associated with childhood cancers (i.e., *TP53*), or if unrelated to the side of the family with the P/LP variant.

## 3 Results

### 3.1 Proband Uptake of Genetic Counselling and Testing

**FIGURE 1:**
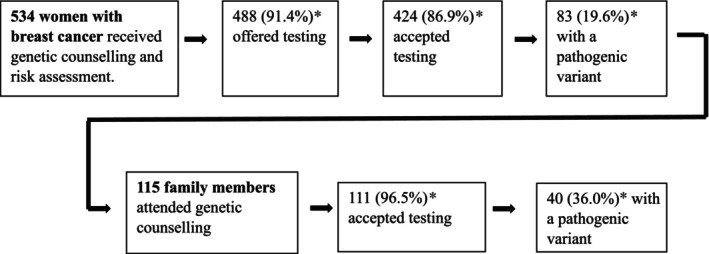
Flow chart of the genetic counseling and testing process for hereditary breast cancer in probands and family members. *Percentages in rows are as a proportion of the previous block.

In the study period, 534 women with a previous or current diagnosis of BC (probands) were seen for pre‐test genetic counselling, of whom 488 (91.4%) were offered genetic testing, and 86.9% (424/488) accepted (Figure 1). Demographic and clinical data for all study participants are shown in Table 3.

Age at diagnosis (*p* < 0.01), the presence of a cancer family history meeting NDOH testing criteria (*p* = 0.05), living > 50 km from the hospital (*p* = 0.02) and subsidisation category (H0 and H1 vs. others) (*p* < 0.01) were included in the multivariate logistic regression analysis. Cancer stage (Stages 0, 1, 2 vs. Stages 3, 4 and recurrence) (*p* = 0.96) and age at consultation (*p* = 0.02) were excluded from the model due to a lack of significance or collinearity with other variables. Testing uptake was found to be associated with younger age at diagnosis, the presence of a family history, and a higher subsidisation level of test costs (Table 4). The impact of test cost on uptake was supported by the finding that 93.3% (42/45) of probands who gave a reason for declining testing named financial concerns.

**TABLE 4:** Logistic regression showing factors impacting acceptance of genetic testing.

Outcome: uptake of testing	Regression coefficient	Std. error	*Z*	*p*	Odds ratio (95% CI)
Intercept	1.88	0.82	2.28	0.02	6.56 (1.33–34.02)
Age at diagnosis	−0.04	0.01	−2.63	0.01	0.96 (0.94–0.99)
Dist. to hospital	−0.59	0.43	−1.36	0.17	0.56 (0.22–1.22)
Family history	0.94	0.37	2.54	0.01	2.57 (1.28–5.55)
Financial subsidy	2.21	0.44	5.07	0.00	9.14 (3.88–21.83)

Abbreviation: CI, confidence interval.

### 3.2 Detection of Pathogenic Variants in Probands

A heterozygous P/LP variant was detected in 19.6% (83/424) of tested probands, with variants in *BRCA1/2* accounting for 78.3% (65/83) (Table S2). The prevalence of P/LP variants in *BRCA1* and *BRCA2* was 5.9% (25/424) and 9.4% (40/424) respectively, with fewer founder variants in *BRCA1* than *BRCA2* (6/424 vs. 32/424, *n* = 424, *p* < 0.001). The detection rate of P/LP variants in other high‐risk susceptibility genes tested was: *PALB2* (4/373, 1.1%), *TP53* (2/373, 0.5%); and in moderate‐risk susceptibility genes: *ATM* (7/372, 1.9%), *CHEK2* (3/372, 0.8%), *RAD51C* (1/321, 0.3%) and *RAD51D* (1/321, 0.3%).

### 3.3 Family Member Genetic Counselling and Testing

Of the 83 probands with a P/LP variant, result feedback was documented in all but one case. Almost half of the probands (45.8% [39/83]) had at least 1 family member receive genetic testing (median: 2, range: 1–11). Only 3.5% (4/115) of family members who attended genetic counselling declined testing. The median time between the first proband consultation and family member consultation was 3.3 months (range: 0.4–20.8 months). Probands reporting more family members diagnosed with cancers (including melanoma, breast, ovarian, prostate, or pancreatic) were more likely to have family members test (OR 1.73, 95% CI 1.16–2.57, *p* < 0.01). No relationship was found between having a family member attend and the age of the proband's cancer diagnosis (*p* = 0.26), the proband's cancer stage (*p* = 0.91), whether the proband lived < 50 km from the hospital (*p* = 0.47), the number of eligible family members (*p* = 0.55), whether the test provider offered free family variant testing (*p* = 0.31), or the proband's financial subsidy category (*p* = 0.75).

Using the family pedigrees collected at the time of consultation, there were 1202 first‐, second‐ and third‐degree relatives eligible for testing (Table 5), of whom 111 (9.2%) were tested. Female family members (OR: 2.0, 95% CI: 1.16–3.43, *p* = 0.01) and first‐degree relatives (OR: 17.1, 95% CI: 5.27–60.48, *p* < 0.001) were significantly more likely to have testing, with 25.4% of female first‐degree relatives receiving cascade testing.

**TABLE 5:** Detection of P/LP variants in eligible family members.

Degree of relationship to proband	Gender	Number of eligible family members	Family members tested (as % of eligible members)	Family members with P/LP variant as proportion of tested (%)
First‐degree	F	228	58 (25.4%)	24/58 (41.4%)
	M	180	24 (13.3%)	9/24 (37.5%)
	Total	408	82 (20.1%)	33/82 (40.2%)
Second‐degree	F	308	24 (7.8%)	6/24 (25%)
	M	271	0	0
	Total	579	24 (4.1%)	6/24 (25%)
Third‐degree	F	125	5 (4.0%)	1/5 (20%)
	M	90	0	0
	Total	215	5 (2.3%)	1/5 (20%)
All	F	661	87 (13.2%)	31/87 (35.6%)
	M	541	24 (4.4%)	9/24 (37.5%)
	Total	1202	111 (9.2%)	40/111 (36.0%)

The overall detection rate for P/LP variants in tested family members was 36.0% (40/111). Of the 40 family members found to carry a P/LP variant, 31 were female and 9 were male, with median ages of 43 (range 20–66) and 35 (range 20–56) years, respectively. P/LP variants were found in the following genes for males: *BRCA1* (6), *BRCA2* (3); and in females: *ATM* (3), *BRCA1* (11), *BRCA2* (14), *PALB2* (3). There was evidence of result feedback in all family members. The identification of test‐positive probands and cascade testing of their family members allowed detection of one test‐positive family member for every 11 probands tested (40/424).

### 3.4 Uptake of Risk‐Reducing Strategies

Most probands with a P/LP variant (64/81, 79.0%) had breast surgery as part of their treatment for BC. Reasons BC surgery was not performed included advanced cancer stage, inoperability, loss to follow‐up, or the proband declining the procedure. Among the probands who underwent BC surgery, 82.8% (53/64) also had surgery performed on the contralateral breast. However, the reason for surgery frequently was undocumented. Of the 50 probands who were eligible for BSO, considering guidelines for the relevant gene, their age and prognosis, 17 (34.0%) had it performed, and 8 (16.0%) said they would consider having it done in the future, although they were already at the recommended age for the procedure.

Of the tested family members, seven had a prior diagnosis of BC, six of whom (85.7%) tested positive for a P/LP variant. As they had previously had surgical treatment, the uptake of RRM was not assessed. However, 4/6 (66.7%) were eligible for risk‐reducing BSO considering their age and the applicable gene guidelines, and two proceeded.

Table 6 outlines risk‐reducing measures undertaken by cancer‐unaffected family members who tested positive for a P/LP variant. As a proportion of those eligible for each intervention, more had RRM (52.6%; 10/19) than mammographic surveillance (30.0%; 3/10) or BSO (15.4%; 2/13). Of those eligible for breast surveillance and/or RRM, 13/20 (65.0%) had one of these interventions. Only one eligible family member had both RRM and BSO procedures done.

**TABLE 6:** Risk‐reducing measures used by female family members without breast cancer.

Risk‐reducing measure	No. eligible for intervention (*n* = 25)^a^	Outcome	No. of participants	Median age and range
Breast surveillance in non‐RRM participants by mammogram and/or ultrasound	10	Performed at least once	3 (30.0%)	37 (36–66)
		Participant reported no breast surveillance done	3 (30.0%)	37 (35–43)
		No record of surveillance^b^	4 (40.0%)	52 (34–63)
Risk‐reducing mastectomy (RRM)	19	RRM	10 (52.6%)	43.5 (33–56)
		Not done	7 (36.8%)	37 (34–66)
		No record of surgery or associated histology^b^	2 (10.5%)	50 (37–63)
Bilateral salpingo‐oophorectomy (BSO)	13	BSO	2 (15.4%)	51.5 (51–52)
		BSO not done	6 (46.2%)	47.5 (35–66)
		No record of surgery or associated histology^b^	5 (38.5%)	43 (36–63)

^a^ Indication for the risk‐reducing measure was determined based on gene guidelines and participant age according to NCCN guidelines (Table 1).

^b^ When no record could be found for surgery or associated histology, it is highly likely that the procedure was not done in the state system, as electronic health records were thoroughly searched for each participant.

## 4 Discussion

In our assessment of the GCT and management path for hereditary BC in a LMIC, we found a high uptake of genetic testing in women with BC, but test cost was a significant factor in determining uptake. Testing in eligible family members was most strongly associated with female gender, being a first‐degree relative, and having multiple family members affected by cancer. Although the uptake of cascade testing was low overall, more than half of eligible test‐positive relatives proceeded with RRM. BSO and mammographic surveillance were less frequently used.

### 4.1 Genetic Counselling and Testing of Probands

The high uptake (86.9%) of genetic testing suggests that it is an acceptable option for most women with BC but may be unaffordable for some. This was indicated by the finding that probands with higher subsidisation of healthcare costs were nine times more likely to test. This probably relates to the fact that all the available genetic tests were expensive: approximately USD125 for founder mutation testing and over USD300 for sequencing of the *BRCA1/2* genes or broader gene panels. The individuals without subsidy, typically referred to as the ‘missing‐middle’ have sufficient household income to disqualify them from assistance but lack the financial resources to pay for medical insurance for genetic testing. This issue is not unique to South Africa. A scoping review of 24 studies on BC testing, all conducted in the USA, found that women without private insurance were not only less likely to be referred for testing but, when referred, were also less likely to proceed with it [40]. This likely results in a substantial segment of the population unable to access testing and therefore ineligible for cancer risk‐reducing measures. Family history was also important as probands with a family history of *BRCA1/2* related cancers were found to be more than twice as likely to proceed with testing.

The high detection rate of P/LP variants (19.6%) in probands likely reflects the relatively strict criteria for offering genetic testing to women with BC in South Africa, as we have discussed elsewhere [41].

### 4.2 Cascade Testing

Uptake of cascade testing in our study was generally low, and it dropped steeply from 20.1% in first‐degree relatives to 4.1% and 2.3% in second‐ and third‐degree relatives, respectively. The uptake of cascade testing in our study was much lower than reported by a recent meta‐analysis of 87 studies, where the uptake of cascade testing was 41% in eligible first‐ and second‐degree relatives [42]. However, studies included in the meta‐analysis were mainly from HIC and often nested in research environments, settings in which better uptake is expected [7].

There are very few publications from LMIC on the uptake of cascade testing for hereditary BC, or hereditary cancer more broadly. Only two studies in the above‐mentioned meta‐analysis were conducted in LMIC as defined by the World Bank (2022)—one in Malaysia on hereditary BC [25] and one in South Africa on Lynch syndrome [24]. The former reported a low uptake of cascade testing, with 78% of probands informing their families but only 11% of relatives coming forward, despite the availability of free testing within a research setting [25]. The authors noted that families expressed concerns regarding ‘cultural taboos about cancer diagnoses, social marginalization and lack of regulatory control of genetic discrimination’. Our study showed a similarly low uptake, but the reasons for this require further exploration. The study on Lynch syndrome showed a contrastingly high uptake of cascade testing, with 97% of siblings and 73.6% of children accepting predictive testing [24]. Like our study, it was set in South Africa, but it described a bespoke research‐based outreach programme to rural areas with high rates of Lynch syndrome. Our study assessed a clinical practice environment covering a healthcare region and therefore appears more representative of the medical mainstream.

The predominance of testing by female family members (77.0%) aligns with findings from a meta‐analysis, which reported that female relatives were almost twice as likely to undergo testing compared to males [42]. This trend is likely attributable to their elevated risk for associated cancers and a greater propensity to utilize health services [43]. Similarly, the meta‐analysis, consistent with our findings, demonstrated that first‐degree relatives were significantly more likely to undergo testing than second‐degree relatives [42]. This may be due to their closer relationship with the proband, which places them at higher risk and facilitates better communication [44]. Higher test uptake was associated with a family history of cancer in both probands and family members. Although not frequently reported in the literature, this finding is intuitive, as individuals from families with significant cancer histories often experience heightened cancer‐related anxiety, which increases compliance with testing and screening [45].

Close to half of test‐positive probands had at least one family member attend genetic counselling, but the number of family members attending varied widely. The reasons for this require further assessment in our setting. Many specific family and contextual factors have also been reported to influence the uptake of testing in at‐risk family members in Western and Asian countries. Described family factors include family support, communication and dynamics, while contextual factors include access to and cost of healthcare services [18]. Of the contextual factors, low income [20], lack of health knowledge [23] and difficulty accessing genetic services [18] are barriers that plausibly impacted many family members in our study.

### 4.3 The Flow From Proband Genetic Counselling to Family Member Testing

The model developed by Offit et al. [17] demonstrated that if all individuals with BC had testing, and there was a 50% to 70% uptake among first‐, second‐ and third‐degree relatives of those identified with a P/LP variant, the majority of carriers in the population could be detected. This is far removed from our study, where (1) we could offer genetic testing only to probands at high risk of a hereditary cancer and (2) cascade testing uptake, even in those at highest risk (female first‐degree relatives) was only 25.7%. In our setting, public health‐level, as opposed to individual‐level, impact from cascade testing will be difficult to achieve.

Instead, we provide real‐world data on the drop‐offs at each stage of GCT in probands and the detection of carrier status in their relatives. The high uptake and detection rate in probands, together with cascade testing uptake that was low but mainly among first‐degree relatives, resulted in the detection of 1 carrier relative being detected for every 13 probands receiving genetic counselling and for every 11 probands receiving genetic testing. This information will facilitate future assessment of the cost–benefit of the GCT programme.

### 4.4 Uptake of Risk‐Reducing Measures

Uptake of mammographic surveillance in family members (30%) was lower than that of RRM, and far lower than found by Metcalfe et al. [16], where uptake was above 96% in all countries offering mammographic surveillance. In contrast, the uptake of RRM by 52.6% of eligible family members with a P/LP variant is above the upper end of the range (4.5%–45%) observed by Metcalfe et al. (2019) across 10 mostly HIC [16]. The impact of RRM on BC‐specific and overall mortality compared to recommended screening is debated in HIC [46]. However, in our setting, where MRI is not widely available and access to advanced cancer therapies is limited, the higher uptake of RRM may be appropriate [47, 48, 49].

In contrast to RRM, the uptake of BSO (15.4%) in eligible women without BC was considerably lower than that found in other studies, which had uptake rates varying from 36.7% to 83.3% [16, 50, 51]. Likewise, the uptake of BSO in probands and family members with BC (35.2%), although higher than in unaffected family members, was half that found in women with BC by Metcalfe et al. [16] (70.7%). It is not clear if this relates to a lack of access or awareness or other reasons. The finding of Makhnoon et al. [50] that higher parity was associated with BSO uptake independent of age suggests that a desire to maintain fertility may be a factor. The low uptake found in our study is concerning as BSO may be more effective in preventing morbidity and mortality in *BRCA1/2* and *PALB2* carriers than RRM [13].

In probands, the available information on risk‐reducing measures was more difficult to interpret than in family members. Contralateral breast surgery in a person with unilateral BC does not necessarily imply that it was done for purposes of risk reduction because of the common practice to offer contralateral breast reduction for cosmetic reasons. The reason for contralateral surgery was most often undocumented, and therefore we could make no inferences from the fact that over 80% of breast surgeries were bilateral. BSO is more likely to be performed to reduce the risk of future cancer, and half of eligible probands either had BSO performed or indicated they would do it in thefuture.

### 4.5 Strengths and Limitations

There were several limitations in this study. Firstly, we relied on clinical records whose completeness was dependent on the consulting clinicians. This was evident in the pedigree data used where a higher number of female family members and first‐degree relatives were counted as being eligible for genetic testing, suggesting that males and more distant relatives were undercounted. However, routine clinical and demographic data was nearly complete, indicating good data quality. This was expected because each electronic data source had proven reliable in clinical practice, and most data points were covered by two sources if required.

The uptake of cascade testing found is likely the ‘best‐case scenario’ because Invitae cascade testing was available in the country at the time, which provided free family member testing if done within a 3‐month period and therefore encouraged expeditious decision‐making about family member testing. Conversely, the uptake of risk‐reducing measures likely represents a minimum estimate. While we expect to have identified most participants who underwent mastectomies and BSOs in the regional state healthcare system given the multiple data sources that we used, we may have missed some who had interventions in the private sector or elsewhere. In addition, the COVID pandemic may have negatively affected uptake; this was not directly assessed, but there was, however, no change in the frequency of cascade testing per year.

A comprehensive assessment of both cascade testing and uptake of risk‐reducing measures would require longer prospective follow‐up, as decisions may take time and eligibility for interventions is age‐dependent. Lastly, the small number of family members detected with P/LP variants meant we did not have sufficient power to investigate factors associated with the uptake of risk‐reducing measures.

## 5 Conclusion

To our knowledge, this is the first assessment of a BC genetic service from detection through to cascade testing and uptake of risk‐reducing strategies in an LMIC country, and it provides a useful framework for further investigations. The high proband test uptake and detection rate suggests that testing is acceptable and relevant. However, limited uptake of both cascade testing and some preventive interventions (BSO and mammography) reduced the effectiveness of the programme. Reasons for the low uptake of cascade testing and variable uptake of risk‐reducing measures should be explored. Comparative data from within and beyond South Africa and a cost–benefit analysis will help guide policy development in our and other LMIC settings.

## Author Contributions

**T. S. Osler:** conceptualization (equal), data curation (equal), formal analysis (lead), investigation (lead), methodology (equal), validation (lead), visualization (lead), writing – original draft (lead), writing – review and editing (lead). **M. Schoeman:** funding acquisition (supporting), investigation (lead), writing – review and editing (equal). **J. Edge:** data curation (supporting), investigation (supporting), writing – review and editing (supporting). **W. J. S. Pretorius:** data curation (equal), investigation (equal), project administration (supporting), writing – review and editing (supporting). **F. H. Rabe:** data curation (supporting), writing – review and editing (supporting). **C. G. Mathew:** funding acquisition (equal), supervision (equal), writing – review and editing (equal). **M. F. Urban:** conceptualization (lead), funding acquisition (lead), methodology (lead), project administration (lead), supervision (lead), validation (equal), visualization (equal), writing – review and editing (lead).

## Ethics Statement

The study was approved by the Human Research Ethics Committees of Stellenbosch University (N22/04/039) and the University of the Witwatersrand (M231195). As the study was a retrospective record review, participant consent was not required. All data were de‐identified. The study adhered to the principles of the Declaration of Helsinki.

## Conflicts of Interest

The authors declare no conflicts of interest.

## Supporting information


Data S1.

## Data Availability

The de‐identified participant data used and analyzed in this study are available from the corresponding author on request.
