# Supplementary material for: Breast Cancer Genetic Services in a South African Setting: Proband Testing, Cascading and Clinical Management
Source: Cancer Med. 2025 Mar 5;14(5):e70743. doi: 10.1002/cam4.70743 (PMC11881015; doi:10.1002/cam4.70743)
Supplement: Supplementary file 1 — Data S1. [file CAM4-14-e70743-s001.docx]

**Supplementary Materials**

**
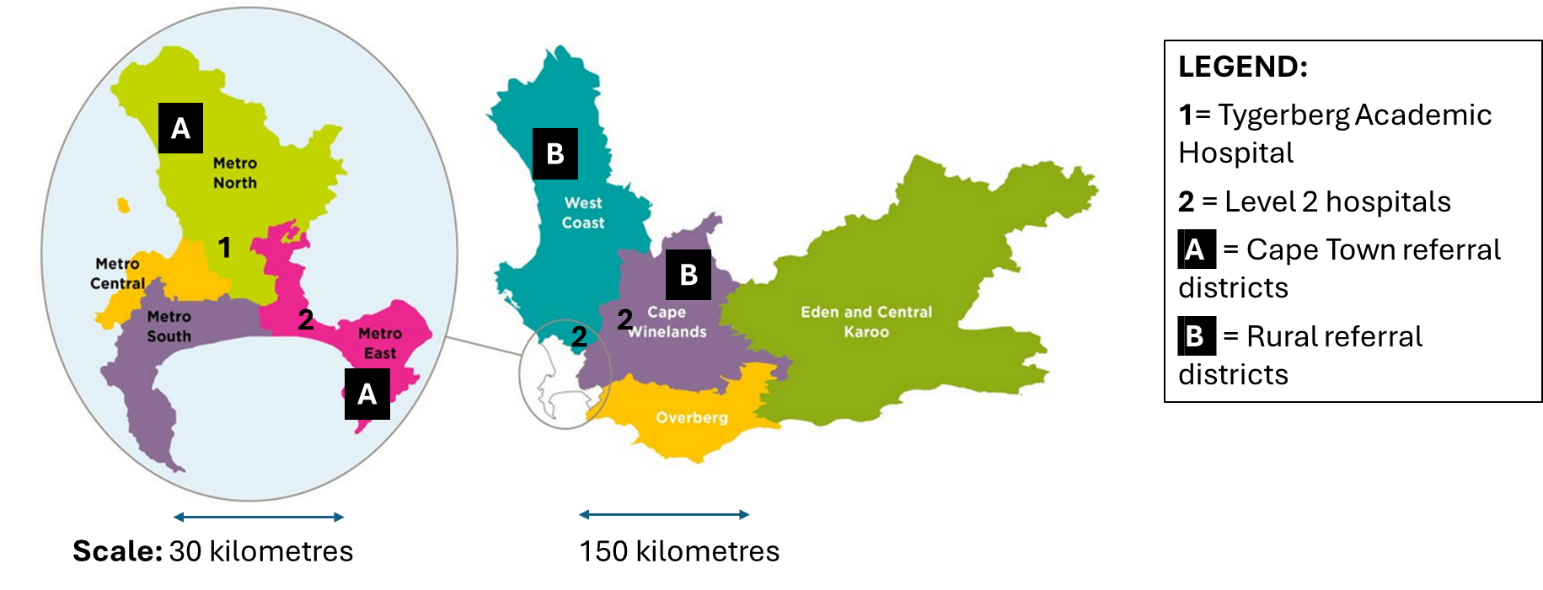
**

**Supplementary Figure 1. Tygerberg Hospital referral region**

^*^Figure modified from https://wcedonline.westerncape.gov.za/contact/districts

**Supplementary Table 1. Founder variants found in *BRCA1* and *BRCA2* in South African populations**

| Ancestry | Founder Variant |
| --- | --- |
| Afrikaner: | NM_007294.3 (*BRCA*1):c.1374del |
|  | NM_007294.3 (*BRCA1*):c.2641G>T |
|  | NM_000059.3 (*BRCA2*):c.7934del |
| Black African: | NM_000059.3 (*BRCA2*):c.5771_5774del |
|  | NM_000059.3 (*BRCA2*):c.582G>A |
| Ashkenazi Jewish: | NM_007294.3 (*BRCA1*): c.68_69del |
|  | NM_000059.3(*BRCA2*):c.5946del |
|  | NM_007294.3 (*BRCA1*):c.5266dup |

**Supplementary Table 2. P/LP variants detected in probands**

| Gene | P/LP variant | Frequency in probands | Founder variant^a^ |
| --- | --- | --- | --- |
| ATM | NM_000051.3:c.5228C>T | 2 |  |
|  | NM_000051.3:c.5279dup | 1 |  |
|  | NM_000051.3:c.6139_6146del | 1 |  |
|  | NM_000051.3:c.7271T>G | 2 |  |
|  | NM_000051.3:c.8307G>A | 1 |  |
| BRCA1 | NM_007294.3:c.1016dup | 1 |  |
|  | NM_007294.3:c.1360_1361del | 1 |  |
|  | NM_007294.3:c.1374del | 1 | Afrikaner |
|  | NM_007294.3:c.1398del | 1 |  |
|  | NM_007294.3:c.181T>G | 1 |  |
|  | NM_007294.3:c.1953_1956del | 2 |  |
|  | NM_007294.3:c.2307_2313del | 1 |  |
|  | NM_007294.3:c.2641G>T | 5 | Afrikaner |
|  | NM_007294.3:c.4484G>T | 1 |  |
|  | NM_007294.3:c.5096G>A | 1 |  |
|  | NM_007294.3:c.5153-1G>A | 1 |  |
|  | NM_007294.3:c.66dup | 3 |  |
|  | NC_000017.11:g.(?_43082404)_(43124096_?)del | 2 |  |
|  | NC_000017.11:g.(?_43070928)_(43124096_?)del | 1 |  |
|  | NM_007294.4:c.5278_5406del | 2 |  |
|  | NC_000017.11:g.(?_43104261)_(43106533_?)del | 1 |  |
| BRCA2 | NM_000059.3:c.3865_3868del | 2 |  |
|  | NM_000059.3:c.3881T>A | 2 |  |
|  | NM_000059.3:c.5771_5774del | 13 | Black African |
|  | NM_000059.3:c.582G>A | 3 | Black African |
|  | NM_000059.3:c.5946del | 1 | Ash. Jew |
|  | NM_000059.3:c.6447_6448dup | 4 |  |
|  | NM_000059.3:c.7558C>T | 1 |  |
|  | NM_000059.3:c.7934del | 12 | Afrikaner |
|  | NM_000059.3:c.8961_8964delGAGT | 1 |  |
|  | NM_000059.3:c.9105T>A | 1 |  |
| CHEK2 | NM_007194.3:c.1100del | 1 |  |
|  | NM_007194.3:c.283C>T | 1 |  |
|  | NM_007194.3:c.779del | 1 |  |
| PALB2 | NM_024675.4:c.2167_2168del | 1 |  |
|  | NM_024675.4:c.2835-1G>C | 3 |  |
| RAD51C | NM_058216.3:c.491_492del | 1 |  |
| RAD51D | NM_002878.4:c.619T>C | 1 |  |
| TP53 | NM_000546.6:c.742C>T | 1 |  |
|  | NM_000546.6:c.831_835del | 1 |  |

^a^ Eight founder variants occur commonly in South Africa:.6 were found in one or more probands and are indicated according to the population in which they were first described. Two not found were Ashkenazi Jewish founders (BRCA1:c.5266dup and BRCA1: c.68_69del).
